# Variation in airborne pollen concentrations among five monitoring locations in a desert urban environment

**DOI:** 10.1007/s10661-018-6738-8

**Published:** 2018-06-25

**Authors:** Tanviben Y. Patel, Mark Buttner, David Rivas, Chad Cross, Dennis A. Bazylinski, Joram Seggev

**Affiliations:** 1 Department of Environmental and Occupational Health, School of Community Health Sciences, University of Nevada Las Vegas, 4505 S. Maryland Pkwy, P. O. Box 453064, Las Vegas, NV 89154 USA; 2 School of Medicine and School of Community Health Sciences, University of Nevada Las Vegas, Las Vegas, NV USA; 3 School of Life Sciences, University of Nevada Las Vegas, Las Vegas, NV USA

**Keywords:** Pollen, Airborne, Allergen, Outdoor air quality, Las Vegas

## Abstract

The urbanization of the Las Vegas Valley has transformed this part of the Mohave Desert into a green oasis by introducing many non-native plant species, some of which are allergenic. Typically, one monitoring station is established per city to obtain pollen counts for an entire metropolitan area. However, variations in pollen concentrations could occur among different microenvironments. The objective of this study is to measure and compare pollen concentrations in five locations in Las Vegas to determine if there are significant differences between microenvironments within the city. Air samples were collected from five sites across the Las Vegas Valley over a 1-year period. Prepared slides were analyzed with a light microscope for pollen grains and converted into airborne pollen concentrations. Mixed model methods were used to determine mean differences. Tree pollen was the greatest contributor to the annual average airborne pollen concentrations (130 grains/m^3^) compared to weeds (6 grains/m^3^) and grass (3 grains/m^3^). The highest peak occurred in March 2016 (9589 total grains/m^3^). There were several differences among sites with respect to concentrations of individual tree species and for total weed and grass concentrations. We observed significant variations in concentration and composition among the five pollen collection stations that were established across the Las Vegas Valley. This study presented new outdoor pollen data for the southwest region of the USA, focused in Las Vegas. The results indicate that more sites and comprehensive monitoring of outdoor allergens are needed to provide accurate information to the community about outdoor air quality conditions.

**Electronic supplementary material:**

The online version of this article (10.1007/s10661-018-6738-8) contains supplementary material, which is available to authorized users.

## Introduction

In the Mojave Desert, there are numerous species of vegetation, but few are native; these include several species of the tree family *Cupressaceae* (Cedar/Juniper), and the genera *Pinus* (Pine) and *Prosopis* (Mesquite). Most species of weeds native to the Mojave Desert belong to the genera *Artemisia* (Sagebrush), *Ambrosia* (Ragweed), and the family *Chenopodiaceae/Amaranthaceae* (Goosefoot/Pigweed) (Holmgren et al. 2010). The urbanization of the Las Vegas Valley, located in a relatively high-altitude area of the Mojave Desert, boomed in the 1960s and 1970s owing to tourism and a nationwide migration. Currently, the population of Las Vegas is approximately 2.1 million (Acevedo et al. 2016). This growth in the population triggered a large increase in housing development and landscaping. Development turned the desert into a green oasis by introducing many non-native plant species, some of which are allergenic, such as *Acer* (Maple), *Fraxinus* (Ash), *Morus* (Mulberry), *Olea* (Olive), *Pinaceae* (Pine), *Platanus* (Sycamore), and *Ulmus* (Elm) (Holmgren et al. 2010).

Pollen allergies are observed during flower blooming and pollination season, and are linked to temperature and wind (Gioulekas et al., 2004; Erkara 2008). Both pollen counts and climate conditions affect the development of allergies (Silverberg et al. 2015). Allergic rhinitis has become more prevalent in developed countries and respiratory illnesses have been linked to pollen allergies (Myszkowska et al. 2012). Previous studies have shown that there is an increase in the incidence of pollen allergies in urban zones (Gonzalo-Garjo et al. 2006). Therefore, measuring pollen concentrations is an important component of outdoor air quality monitoring.

Typically, the National Allergy Bureaus (NAB) uses one or two monitoring stations per city to provide pollen counts for an entire metropolitan area (National Allergy Bureau 2017) However, variations in pollen concentrations could occur among diverse microenvironments resulting in differences in human exposure to allergenic pollens. The objective of this study was to measure and compare pollen concentrations in five locations in Las Vegas to determine if and what kinds of differences occur between microenvironments within the city.

## Methods

### Air sampling, collection, and analysis

Air samples were collected from sites across the Las Vegas Valley using a Burkard spore trap (Burkard Manufacturing Company, Rickmansworth, Hertfordshire, England). Samples were collected from April 7th 2015 to April 6th 2016. Either 24-h or 7-day samples were collected. For 24-h samples, a 3″ × 1″ plain glass microscope slide was coated with a thin film of high vacuum grease (Dow Corning Corporation, Midland, Michigan) and inserted into the sampler. Airborne particles were impacted onto the slide for 24 h at an air flow rate of 10 l per minute (Levetin et al. 2000). The sample was collected daily and the slide was placed onto a slide warmer for 10 min (Electron Microscopy Sciences®, Hatfield, Pennsylvania). A coverslip was then applied with a few drops of glycerin jelly stained with basic fuchsin (Levetin et al. 2000). For 7-day samples, a strip of Melinex® (New Berlin, Wisconsin) tape was fixed to the 7-day sampler drum and coated with a thin film of grease. The sampler drums were changed weekly and the tapes were cut into 48-mm segments, representing 24-h increments. The cut sections were adhered to a microscope slide with a 10% Gelvatol solution (Robert E. Esch, Lenoir, North Carolina) and allowed to dry for 10 min. Coverslips were then applied with a few drops of glycerin jelly stained with basic fuchsin. All prepared slides were analyzed with a light microscope at a total magnification of × 400 for pollen grains. A single longitudinal traverse was counted according to NAB protocols (Levetin et al. 2000). Pollen grains were identified, if possible, and counts were converted into airborne concentrations and expressed as pollen grains/m^3^ (Khattab and Levetin 2008).

### Study site

**Fig. 1:**
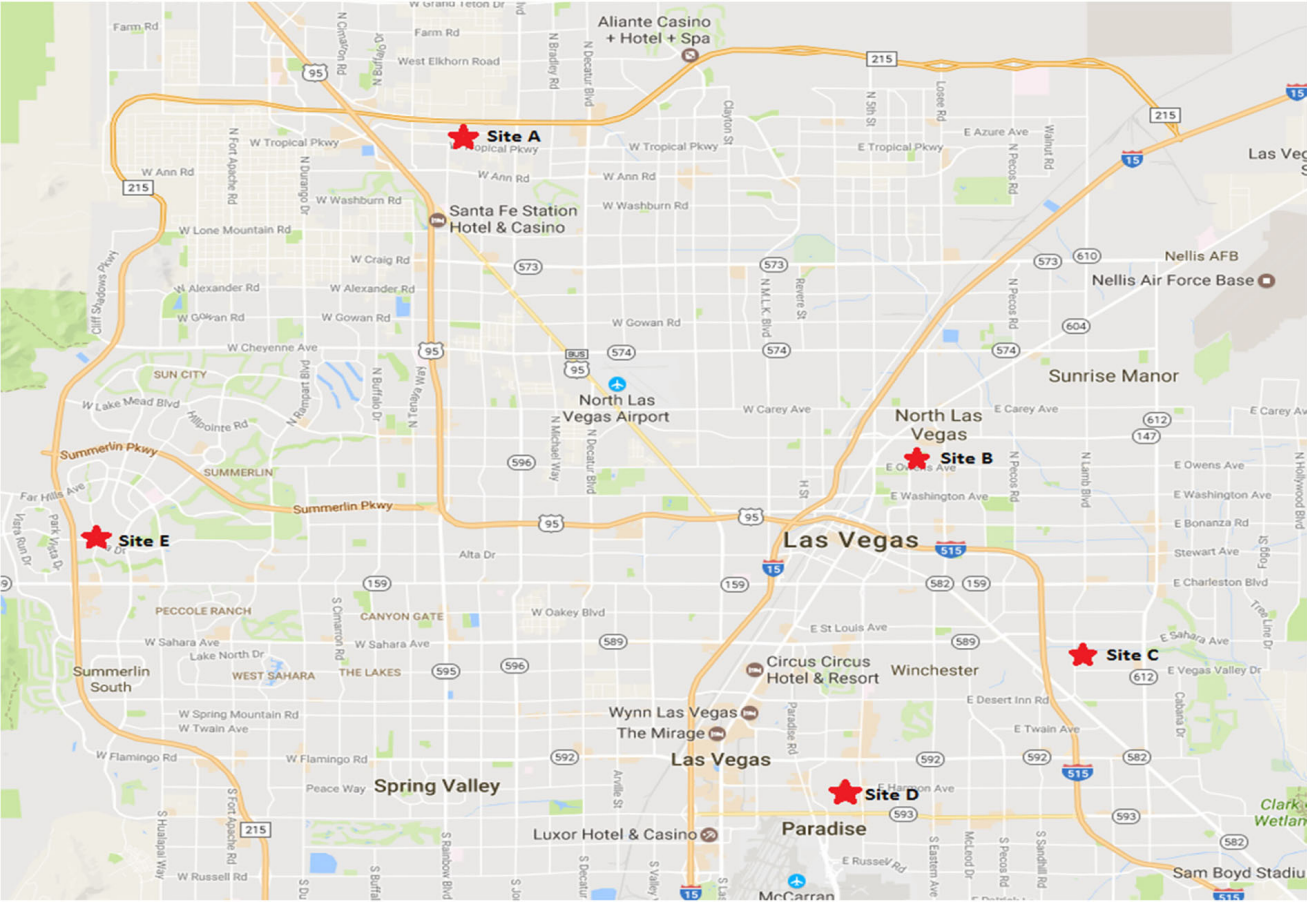
Map of airborne pollen collection sites in Las Vegas, NV (North ↑)

Five monitoring locations were established in Las Vegas (Fig. 1). Sites A, C, and E are located in suburban, newer developed areas in the city close to housing and small roads. Sites B and D are located in densely packed urban areas, in older parts of the city close to busy roads. Samplers were mounted onto single-story rooftop structures for sites A, B, C, and E. The Site D sampler was mounted on a three-story building rooftop.

In the Las Vegas Valley, temperatures range from a monthly average high of 40 °C to an average low of 3 °C (Table 1). The relative humidity ranges from 17 to 30% during the spring season and the driest days are typically in June, with a relative humidity of 13%. The average precipitation is 101 to 127 mm per year. The typical wind speed ranges from 0 to 4 m per second (mps), with average wind gusts of 12 mps.

**Table 1:** Meteorological data for Las Vegas, NV, in 2015 and 2016 (Source: timeanddate.com and wunderground.com)

Year	Month	Mean high and low temperature (°C)	Mean relative humidity (%)	Total precipitation (mm)	Mean wind speed/gusts (mps)
2015	April	27/13	17	6.6	5/11
	May	29/18	25	6.1	4/10
	June	40/27	13	0.0	5/11
	July	38/27	21	4.8	4/11
	August	40/28	21	17.3	2/9
	September	37/24	22	0.5	3/9
	October	29/18	36	29.5	3/10
	November	18/7	31	6.1	3/12
	December	13/3	35	0.3	3/11
2016	January	14/4	46	11.7	2/11
	February	21/8	29	2.3	3/12
	March	24/12	24	0.0	4/10
	April	26/14	30	57.4	4/11

### Statistical analysis

A Shapiro-Wilk test and observation of skewness and kurtosis measures were used to determine if the data were normally distributed. For the analyses that followed, all data were log-transformed prior to analysis to meet the assumptions of the modeling approach. A mixed model analysis was used to assess potential differences among locations and months, while treating measurements as repeated factors. The mixed model approach allowed for inclusion of both location and time, and additionally provided a means to account for an auto-regressive time lag in the data, which was important owing to the temporal nature of the data collection. A planned post-hoc analysis based on marginal mean differences was used to determine differences among locations and months when the overall model suggested differences in main effects. The mixed model is generally more robust than the repeated measures ANOVA.

## Results

### Total pollen

**Fig. 2:**
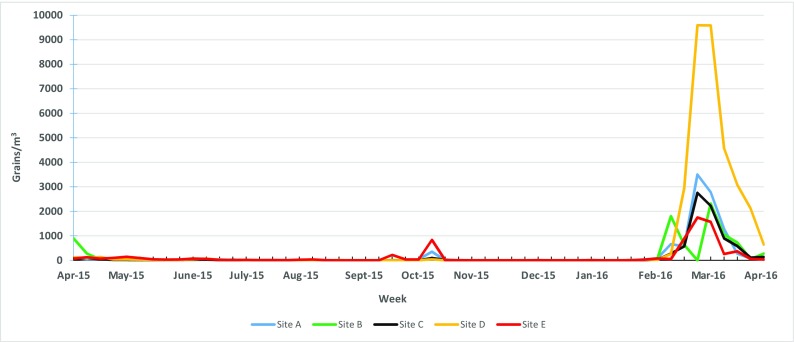
Weekly maximum total tree pollen concentrations from April 2015 to April 2016

Tree pollen was by far the greatest contributor to the annual average airborne pollen concentrations (mean = 130 grains/m^3^) compared to weeds (mean = 6 grains/m^3^) and grass (mean = 3 grains/m^3^). The primary tree pollen season occurred in the months of February to June. The largest peak was at Site D in March 2016 (9589 total grains/m^3^) (Fig. 2). The second largest peak was at Site A in March 2016 (3500 total grains/m^3^). Overall, when total tree pollen data were combined, there were no significant differences among the five locations for annual average tree pollen concentrations (*p* > 0.05). However, there were significant differences by month in total tree pollen concentrations, with March being statistically greater than all other months (*p* ≤ 0.001; Supplemental Table 1).

**Fig. 3:**
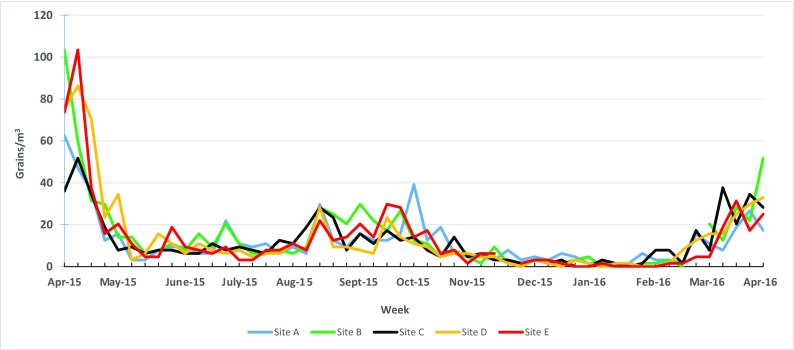
Weekly maximum total weed pollen concentrations from April 2015 to April 2016

Weed pollen concentrations were the highest during the spring months of March to May and the fall months of August to October. Weed pollen at Sites B and D had the highest peaks in April (104 grains/m^3^) (Fig. 3). There were differences among the sites for total weed concentrations. For weed pollen, Sites A, B, and C were all statistically greater than Sites D and E (*p* < 0.05). Sites A and E had the largest mean difference in total weed pollen concentrations (log mean difference = 0.093 grains/m^3^).

**Fig. 4:**
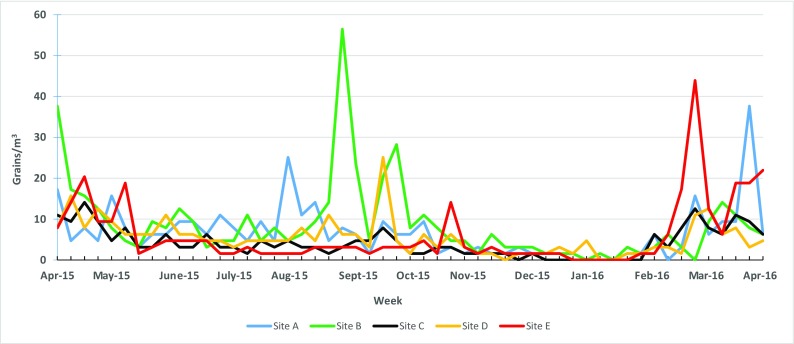
Weekly maximum total grass pollen concentrations from April 2015 to April 2016

Grass pollen concentrations were consistent and similar between the months of March and November. The highest peak was at Site B in September (57 grains/m^3^). Site D had the second highest peak during the month of March (44 grains/m^3^) (Fig. 4). Total grass concentrations were significantly greater in Site A compared with C (log mean difference = 0.091 grains/m^3^, *p* = 0.002) and Site A compared with D (log mean difference = 0.080 grains/m^3^, *p* = 0.007). Site B concentrations were significantly greater than all other sites (*p*  <0.001). Sites B and C had the greatest mean difference (log mean difference = 0.221 grains/m^3^, *p*  < 0.001).

At various times during the year, high concentrations were observed for total tree, grass, and weed pollen, according to the NAB criteria (National Allergy Bureau 2017).

### Individual tree pollen species

There were several differences observed among sites with respect to individual pollen species (Supplemental Fig. 1 a–e). At Site E, there was a large peak in *Acer* pollen concentrations during the month of May (138 grains/m^3^). Site D had the largest spike of *Morus* in March 2016 (8820 grains/m^3^). Site B had the highest peak in *Olea* concentration in April 2015 (591 grains/m^3^). At Site D, the highest peak of *Platanus* was in March (795 grains/m^3^). *Ulmus* had a large peak in October at all sites, with the largest concentration at Site E (834 grains/m^3^).

There were significant differences among sites for several individual tree pollen species (Table 2; Supplemental Fig. 1a–e). Statistically, there was a difference in *Acer* pollen concentrations among Sites A, C, D, and E, with the largest mean difference between Sites E and A (log mean difference = 0.116 grains/m^3^, *p* = 0.005). *Morus* showed the greatest difference between Sites D and E (log mean difference = 0.360 grains/m^3^, *p* = 0.051). *Olea* concentrations were significantly different at Sites A, B, C, and D, with the greatest mean difference between Sites D and A (log mean difference = 0.086 grains/m^3^, *p* = 0.007). *Platanus* concentrations were different at Site D compared to all other sites, with the greatest mean difference between Sites D and B (log mean difference = 0.112 grains/m^3^, *p* = 0.017). It should be noted that Site B had missing data due to a sampler malfunction from 2/24/16 to 3/8/16.

**Table 2:** Comparison of airborne pollen concentrations among five sampling locations for various species of trees (only significant differences are shown)

Pollen type	Location (log mean)	Location (log mean)	Log mean difference	*p* value	95% Confidence interval	
					Lower bound	Upper bound
*Acer* (Maple)	A (0.110)	D (0.210)	− 0.099	0.015	− 0.179	− 0.020
	A (0.110)	E (0.230)	− 0.116	0.005	− 0.196	− 0.037
	C (0.150)	D (0.210)	− 0.083	0.042	− 0.163	− 0.003
*Fraxinus* (Ash)	No significant difference among locations					
*Morus* (Mulberry)	D (0.540)	E (0.170)	0.360	0.051	− 0.001	0.722
*Olea europea* (Olive)	A (0.076)	B (0.156)	− 0.080	0.012	− 0.143	− 0.018
	A (0.076)	D (0.162)	− 0.086	0.007	− 0.148	− 0.024
	B (0.156)	C (0.088)	0.067	0.035	0.005	0.130
	C (0.088)	D (0.162)	− 0.073	0.021	− 0.135	− 0.011
*Pinaceae* (Pine)	No significant difference among locations					
*Platanus* (Sycamore)	B (0.044)	D (0.156)	− 0.112	0.017	− 0.204	− 0.021
	C (0.057)	D (0.156)	− 0.099	0.034	− 0.191	− 0.008
*Ulmus* (Elm)	No significant difference among locations					

## Discussion

The National Allergy Bureau generally provides data for one or two collection stations in a metropolitan area to quantify the dominant allergenic pollen species. However, spatial theories have concluded that an air sampler will collect pollen within only 2 km of the source (McLauchlan et al. 2011). Therefore, variety in vegetation in an urban area would be expected to produce variation in airborne pollen. We observed significant variations in concentration and composition among the five pollen collection stations that were established across the Las Vegas Valley.

When total tree pollen concentrations were averaged over the entire year, there were no significant differences observed between the sites. These results were similar to a study done in Ohio during 2002 that showed no variation in total tree pollen between two sites, one suburban and one urban (White et al. 2005). A similar study in New York City collected samples with Tauber traps at 45 collection sites. Their results showed total tree pollen concentrations varied across the city; concentrations ranged from 2942 to 17,463 grains/cm^2^ (Weinberger et al. 2016). A study in Istanbul, Turkey, showed differences between two sites, with the largest peak seen in April (3797 versus 2715 grains/m^3^) (Celenk et al. 2010).

Total mean weed concentrations varied among the sites in Las Vegas, with the highest peak of 104 grains/m^3^ in April. These results were similar to a study in Istanbul that showed differences in two sites for weed pollen, with the highest peak of 741 grains/m^3^ in September (Celenk et al. 2010). Total grass pollen concentrations showed variations among four of the sites in Las Vegas. A study in London that sampled 14 sites showed differences in grass pollen concentrations between 44 and 77% (Emberlin and Norris-Hill 1991).

Individually, there were several differences among tree pollen species. For example, *Acer* pollen concentrations were higher at Site E compared to all other sites. *Olea* and *Platanus* had their highest concentrations at Site D. A study in Cordoba, Spain, showed the pollen index of *Olea* was higher at their south-west sample site compared to the north-eastern site (Velasco-Jiménez et al., 2013). A similar study with three portable sites and one fixed site showed significant differences among *Olea* at the four sites (Gonzalo-Garjo et al. 2006). Interestingly, ragweed pollen was observed in higher concentrations in spring months than in the fall in Las Vegas, whereas it is typically present only in the fall in other parts of the USA (Frenz et al. 1995).

The variability in pollen concentrations could be explained by the geography and landscaping practices near the sampling sites. Sites A and E are in suburban, residential areas, while Sites B, C, and D are located in densely populated urban areas with older vegetation. The higher weed pollen concentration at Site A might be due to the developing residential area along the outer edge of the city. Celenk et al. (2010) concluded their variation in tree and weed concentrations were due to differences in surrounding vegetation at the sampling sites.

Site D is the official NAB sampling site that is used to report the concentration of pollen for the city of Las Vegas. The concentrations at that site are displayed for the pollen forecast of the day. The public health implication is that when concentrations of certain pollen species are low at Site D they may be higher or lower at other sites located in different parts of the city, which could lead to inaccurate allergen reports. For example, in October at Site E, the mean for *Ulmus* pollen concentrations was much higher compared to Site D. In February, the mean of *Platanus* for Site A was greater than Site D. March had the greatest concentration of *Morus* at Site D compared to all of the other sites. Historically, *Morus* and *Olea* are pollinated later in March and April, respectively, than was observed in this study (unpublished data). Whether the earlier pollination for these species is a trend related to climate change is unknown.

The NAB has established a standard to determine the concentration range that could elicit an allergic reaction in most people. For tree pollen, concentrations above 15 grains/m^3^ of air, for grass, concentrations above 5 grains/m^3^, and for weeds, concentrations above 10 grains/m^3^ are considered high environmental concentrations (National Allergy Bureau 2017). However, an arid climate tends to decrease the prevalence of allergic rhinitis, whereas higher air temperatures and pollen counts tend to increase the prevalence of allergy in children (Silverberg et al. 2015). Airborne pollen concentrations in Las Vegas exceeded NAB standard concentrations several times during the year at the various sites. Therefore, this finding has potential public health significance.

The primary limitation of our study is having only a single year of data, which provides limited information about seasonal consistency and long-term temporal patterns of pollen in this geographic area. Additionally, having only five sites may not provide a complete spatial picture of airborne pollen variation around the Las Vegas Valley.

The data obtained from the five stations established in Las Vegas showed that there are significant variations in airborne pollen concentrations among the sites. The results indicate that more sites and comprehensive monitoring of outdoor allergens are needed to provide accurate information to the community about outdoor air quality conditions. This study presented new outdoor pollen data for the southwest region of the USA, focused in the Las Vegas Valley. Pollen concentrations should continue to be monitored over several years to establish seasonal trends and variability within the microenvironments in the Las Vegas Valley.

## Electronic supplementary material

ESM 1 (PDF 815 kb)
