# Supplementary material for: Variation in airborne pollen concentrations among five monitoring locations in a desert urban environment
Source: Environ Monit Assess. 2018 Jun 25;190(7):424. doi: 10.1007/s10661-018-6738-8 (PMC6018573; doi:10.1007/s10661-018-6738-8)
Supplement: Supplementary file 1 — (PDF 815 kb) [file 10661_2018_6738_MOESM1_ESM.pdf]

Supplementary Material

Variation in Airborne Pollen Concentrations Among Five Monitoring Locations in a Desert Urban Environment.

Authors:

Tanviben Y. Patel, PhD - School of Community Health Sciences, University of Nevada Las Vegas.

Mark Buttner, PhD - School of Community Health Sciences, University of Nevada Las Vegas.

David Rivas, BS - School of Community Health Sciences, University of Nevada Las Vegas.

Chad Cross, PhD, PStat® – School of Medicine and School of Community Health Sciences, University of Nevada, Las Vegas.

Dennis A. Bazylinski, PhD - School of Life Sciences, University of Nevada Las Vegas

Joram Seggev, MD, FAAAAI, FACAAI - School of Community Health Sciences, University of Nevada Las Vegas

Corresponding Author:

Dr. Mark Buttner
Department of Environmental and Occupational Health
School of Community Health Sciences, University of Nevada, Las Vegas
4505 S. Maryland Pkwy, Box 453064, Las Vegas, NV 89154
Phone: 702-895-1418 Fax: 702-895-5184 E-mail: mark.buttner@unlv.edu

Acknowledgments

This study was funded by the Clark County School District, State of Nevada.

Supplementary Table 1: Monthly comparison of tree pollen concentrations among all of the five sites.

Month (Log Mean ± SE)		Log Mean Difference	Std. Error	df	P-value[a]	95% Confidence Interval for Difference [b]	
						Lower Bound	Upper Bound
1 vs. (0.390 ± 0.099)	2	-1.060[*]	0.106	333.307	<0.001	-1.268	-0.852
	3	-1.880[*]	0.114	212.347	<0.001	-2.105	-1.655
	4	-1.268[*]	0.112	200.442	<0.001	-1.49	-1.047
	5	-0.757[*]	0.115	185.521	<0.001	-0.984	-0.53
	6	-0.766[*]	0.116	184.969	<0.001	-0.994	-0.538
	7	-0.236[*]	0.115	184.026	0.042	-0.463	-0.008
	8	0.122	0.115	182.939	0.289	-0.105	0.349
	9	0.191	0.116	186.639	0.099	-0.037	0.419
	10	-0.168	0.115	190.249	0.145	-0.394	0.058
	11	0.221	0.114	220.263	0.054	-0.004	0.445
	12	0.251[*]	0.104	317.367	0.016	0.047	0.455
2 vs. (1.450 ± 0.102)	1	1.060[*]	0.106	333.307	<0.001	0.852	1.268
	3	-0.820[*]	0.108	301.169	<0.001	-1.033	-0.607
	4	-0.209	0.114	226.395	0.068	-0.433	0.015
	5	0.303[*]	0.117	194.831	0.011	0.072	0.534
	6	0.294[*]	0.118	190.497	0.014	0.061	0.527
	7	0.824[*]	0.118	188.568	<0.001	0.592	1.056
	8	1.182[*]	0.118	187.037	<0.001	0.95	1.414
	9	1.251[*]	0.118	189.471	<0.001	1.018	1.484
	10	0.892[*]	0.118	188.696	<0.001	0.66	1.124
	11	1.281[*]	0.118	200.253	<0.001	1.048	1.513
	12	1.311[*]	0.115	220.957	<0.001	1.083	1.538
3 vs. (2.270 ± 0.100)	1	1.880[*]	0.114	212.347	<0.001	1.655	2.105
	2	0.820[*]	0.108	301.169	<0.001	0.607	1.033
	4	0.611[*]	0.103	332.426	<0.001	0.41	0.813
	5	1.123[*]	0.114	215.285	<0.001	0.899	1.347
	6	1.114[*]	0.116	194.157	<0.001	0.885	1.343
	7	1.644[*]	0.116	187.762	<0.001	1.416	1.873

Month (Log Mean ± SE)		Log Mean Difference	Std. Error	df	P-value[a]	95% Confidence Interval for Difference [b]	
						Lower Bound	Upper Bound
	8	2.002*	0.116	185.01	<0.001	1.774	2.23
	9	2.071*	0.116	186.927	<0.001	1.842	2.301
	10	1.712*	0.116	184.761	<0.001	1.483	1.94
	11	2.101*	0.117	191.029	<0.001	1.871	2.331
	12	2.131*	0.115	191.716	<0.001	1.903	2.358
4 vs. (1.659 ± 0.096)	1	1.268*	0.112	200.442	<0.001	1.047	1.49
	2	0.209	0.114	226.395	0.068	-0.015	0.433
	3	-0.611*	0.103	332.426	<0.001	-0.813	-0.41
	5	0.512*	0.102	330.199	<0.001	0.311	0.712
	6	0.502*	0.111	229.32	<0.001	0.283	0.722
	7	1.033*	0.112	203.794	<0.001	0.811	1.254
	8	1.391*	0.113	195.894	<0.001	1.169	1.613
	9	1.460*	0.113	196.725	<0.001	1.236	1.683
	10	1.100*	0.113	193.802	<0.001	0.878	1.323
	11	1.489*	0.113	199.427	<0.001	1.265	1.713
	12	1.519*	0.113	195.604	<0.001	1.297	1.741
5 vs. (1.47± 0.099)	1	0.757*	0.115	185.521	<0.001	0.53	0.984
	2	-0.303*	0.117	194.831	0.011	-0.534	-0.072
	3	-1.123*	0.114	215.285	<0.001	-1.347	-0.899
	4	-0.512*	0.102	330.199	<0.001	-0.712	-0.311
	6	-0.009	0.104	326.59	0.931	-0.214	0.196
	7	0.521*	0.113	219.012	<0.001	0.299	0.744
	8	0.879*	0.114	192.8	<0.001	0.653	1.105
	9	0.948*	0.115	188.889	<0.001	0.721	1.176
	10	0.589*	0.115	184.905	<0.001	0.362	0.815
	11	0.978*	0.116	189.556	<0.001	0.749	1.206
	12	1.008*	0.115	184.986	<0.001	0.781	1.234
6 vs. (1.156 ± 0.100)	1	0.766*	0.116	184.969	<0.001	0.538	0.994
	2	-0.294*	0.118	190.497	0.014	-0.527	-0.061
	3	-1.114*	0.116	194.157	<0.001	-1.343	-0.885
	4	-0.502*	0.111	229.32	<0.001	-0.722	-0.283

Month (Log Mean ± SE)		Log Mean Difference	Std. Error	df	P-value[a]	95% Confidence Interval for Difference [b]	
						Lower Bound	Upper Bound
	5	0.009	0.104	326.59	0.931	-0.196	0.214
	7	0.530*	0.104	327.189	<0.001	0.325	0.735
	8	0.888*	0.114	217.92	<0.001	0.664	1.112
	9	0.957*	0.116	195.829	<0.001	0.729	1.186
	10	0.598*	0.116	187.072	<0.001	0.37	0.826
	11	0.987*	0.116	190.471	<0.001	0.757	1.216
	12	1.017*	0.116	185.431	<0.001	0.789	1.245
7 vs. (0.626 ± 0.099)	1	0.236*	0.115	184.026	0.042	0.008	0.463
	2	-0.824*	0.118	188.568	<0.001	-1.056	-0.592
	3	-1.644*	0.116	187.762	<0.001	-1.873	-1.416
	4	-1.033*	0.112	203.794	<0.001	-1.254	-0.811
	5	-0.521*	0.113	219.012	<0.001	-0.744	-0.299
	6	-0.530*	0.104	327.189	<0.001	-0.735	-0.325
	8	0.358*	0.104	321.31	0.001	0.154	0.562
	9	0.427*	0.114	219.76	<0.001	0.203	0.651
	10	0.068	0.115	192.475	0.557	-0.159	0.294
	11	0.456*	0.116	191.334	<0.001	0.228	0.685
	12	0.487*	0.115	184.996	<0.001	0.26	0.714
8 vs. (0.268 ± 0.099)	1	-0.122	0.115	182.939	0.289	-0.349	0.105
	2	-1.182*	0.118	187.037	<0.001	-1.414	-0.95
	3	-2.002*	0.116	185.01	<0.001	-2.23	-1.774
	4	-1.391*	0.113	195.894	<0.001	-1.613	-1.169
	5	-0.879*	0.114	192.8	<0.001	-1.105	-0.653
	6	-0.888*	0.114	217.92	<0.001	-1.112	-0.664
	7	-0.358*	0.104	321.31	0.001	-0.562	-0.154
	9	0.069	0.104	324.531	0.507	-0.136	0.274
	10	-0.290*	0.113	215.221	0.011	-0.513	-0.068
	11	0.099	0.115	196.295	0.394	-0.129	0.326
	12	0.129	0.115	185.115	0.264	-0.098	0.355
	1	-0.191	0.116	186.639	0.099	-0.419	0.037
	2	-1.251*	0.118	189.471	<0.001	-1.484	-1.018

Month (Log Mean ± SE)		Log Mean Difference	Std. Error	df	P-value[a]	95% Confidence Interval for Difference [b]	
						Lower Bound	Upper Bound
9 vs. (0.199 ± 0.100)	3	-2.071*	0.116	186.927	<0.001	-2.301	-1.842
	4	-1.460*	0.113	196.725	<0.001	-1.683	-1.236
	5	-0.948*	0.115	188.889	<0.001	-1.176	-0.721
	6	-0.957*	0.116	195.829	<0.001	-1.186	-0.729
	7	-0.427*	0.114	219.76	<0.001	-0.651	-0.203
	8	-0.069	0.104	324.531	0.507	-0.274	0.136
	10	-0.359*	0.104	324.195	0.001	-0.564	-0.154
	11	0.029	0.114	224.23	0.797	-0.196	0.255
	12	0.06	0.115	193.598	0.606	-0.168	0.287
10 vs. (0.558 ± 0.099)	1	0.168	0.115	190.249	0.145	-0.058	0.394
	2	-0.892*	0.118	188.696	<0.001	-1.124	-0.66
	3	-1.712*	0.116	184.761	<0.001	-1.94	-1.483
	4	-1.100*	0.113	193.802	<0.001	-1.323	-0.878
	5	-0.589*	0.115	184.905	<0.001	-0.815	-0.362
	6	-0.598*	0.116	187.072	<0.001	-0.826	-0.37
	7	-0.068	0.115	192.475	0.557	-0.294	0.159
	8	0.290*	0.113	215.221	0.011	0.068	0.513
	9	0.359*	0.104	324.195	0.001	0.154	0.564
	11	0.389*	0.104	329.35	<0.001	0.184	0.594
	12	0.419*	0.113	215.184	<0.001	0.196	0.642
11 vs. (0.170 ± 0.100)	1	-0.221	0.114	220.263	0.054	-0.445	0.004
	2	-1.281*	0.118	200.253	<0.001	-1.513	-1.048
	3	-2.101*	0.117	191.029	<0.001	-2.331	-1.871
	4	-1.489*	0.113	199.427	<0.001	-1.713	-1.265
	5	-0.978*	0.116	189.556	<0.001	-1.206	-0.749
	6	-0.987*	0.116	190.471	<0.001	-1.216	-0.757
	7	-0.456*	0.116	191.334	<0.001	-0.685	-0.228
	8	-0.099	0.115	196.295	0.394	-0.326	0.129
	9	-0.029	0.114	224.23	0.797	-0.255	0.196
	10	-0.389*	0.104	329.35	<0.001	-0.594	-0.184
	12	0.03	0.104	329.567	0.773	-0.175	0.235
	1	-0.251*	0.104	317.367	0.016	-0.455	-0.047

Month (Log Mean ± SE)		Log Mean Difference	Std. Error	df	P-value[a]	95% Confidence Interval for Difference [b]	
						Lower Bound	Upper Bound
12 vs. (0.139 ± 0.099)	2	-1.311*	0.115	220.957	<0.001	-1.538	-1.083
	3	-2.131*	0.115	191.716	<0.001	-2.358	-1.903
	4	-1.519*	0.113	195.604	<0.001	-1.741	-1.297
	5	-1.008*	0.115	184.986	<0.001	-1.234	-0.781
	6	-1.017*	0.116	185.431	<0.001	-1.245	-0.789
	7	-0.487*	0.115	184.996	<0.001	-0.714	-0.26
	8	-0.129	0.115	185.115	0.264	-0.355	0.098
	9	-0.06	0.115	193.598	0.606	-0.287	0.168
	10	-0.419*	0.113	215.184	<0.001	-0.642	-0.196
	11	-0.03	0.104	329.567	0.773	-0.235	0.175

Based on estimated marginal means

*** The mean difference is significant at the .05 level.**

a. Dependent Variable: logPollen.

b. Adjustment for multiple comparisons: Fisher's LSD

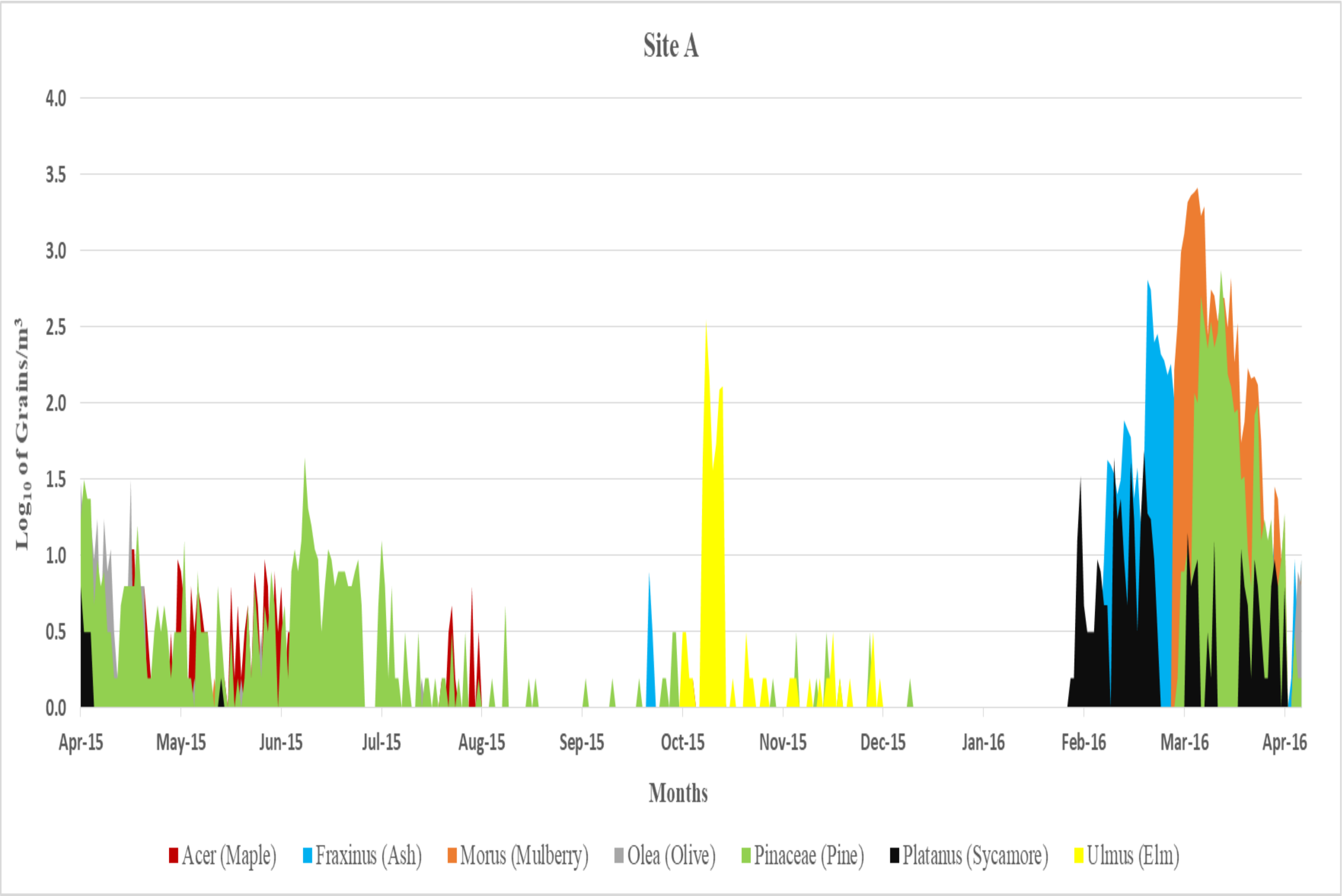

Supplementary Figure 1a. Variation in Tree Pollen for Site A from April 2015 – April 2016.

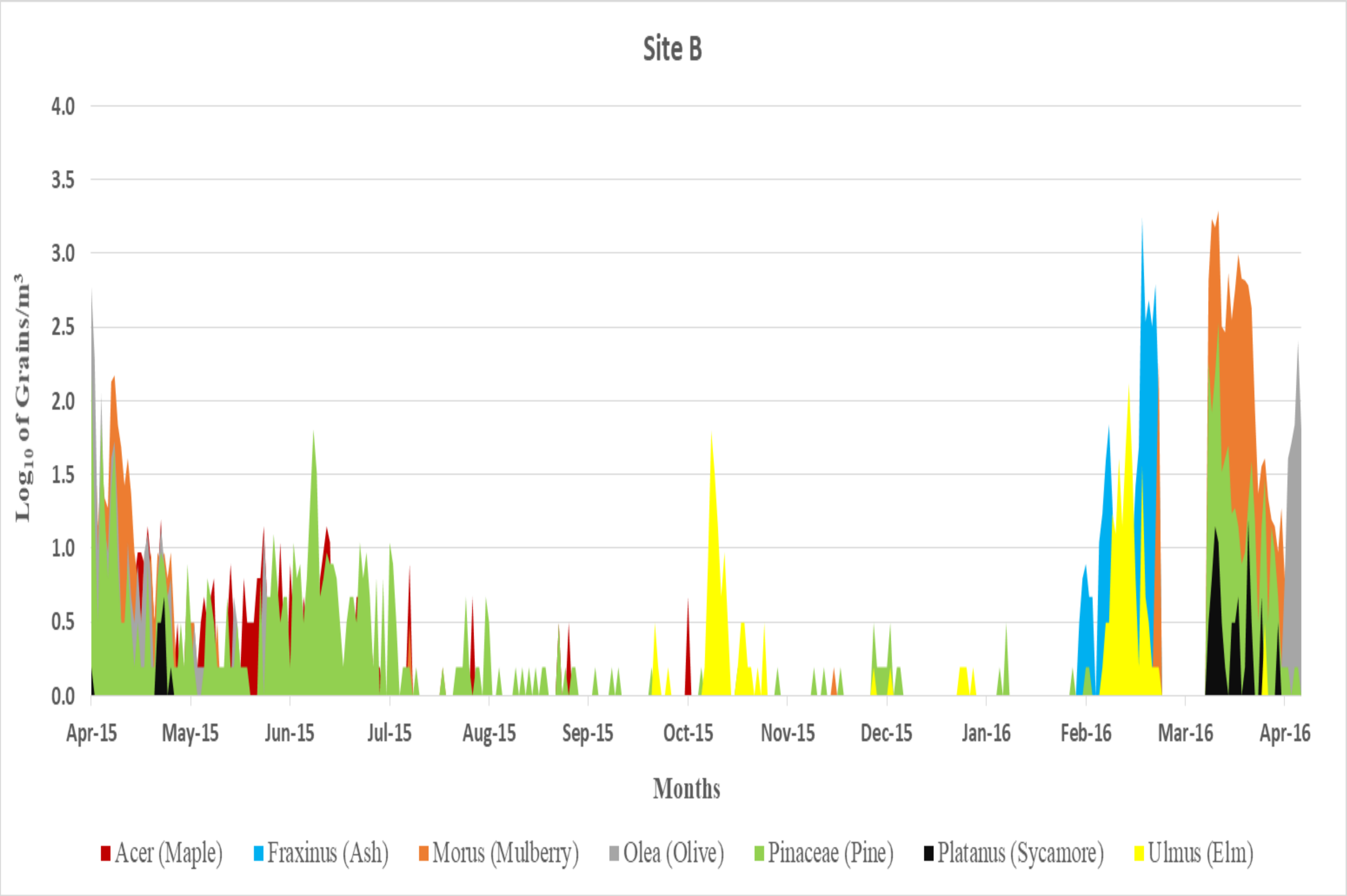

Supplementary Figure 1b. Variation in Tree Pollen for Site B from April 2015 – April 2016 (Missing data from 2/24/16 – 3/8/16).

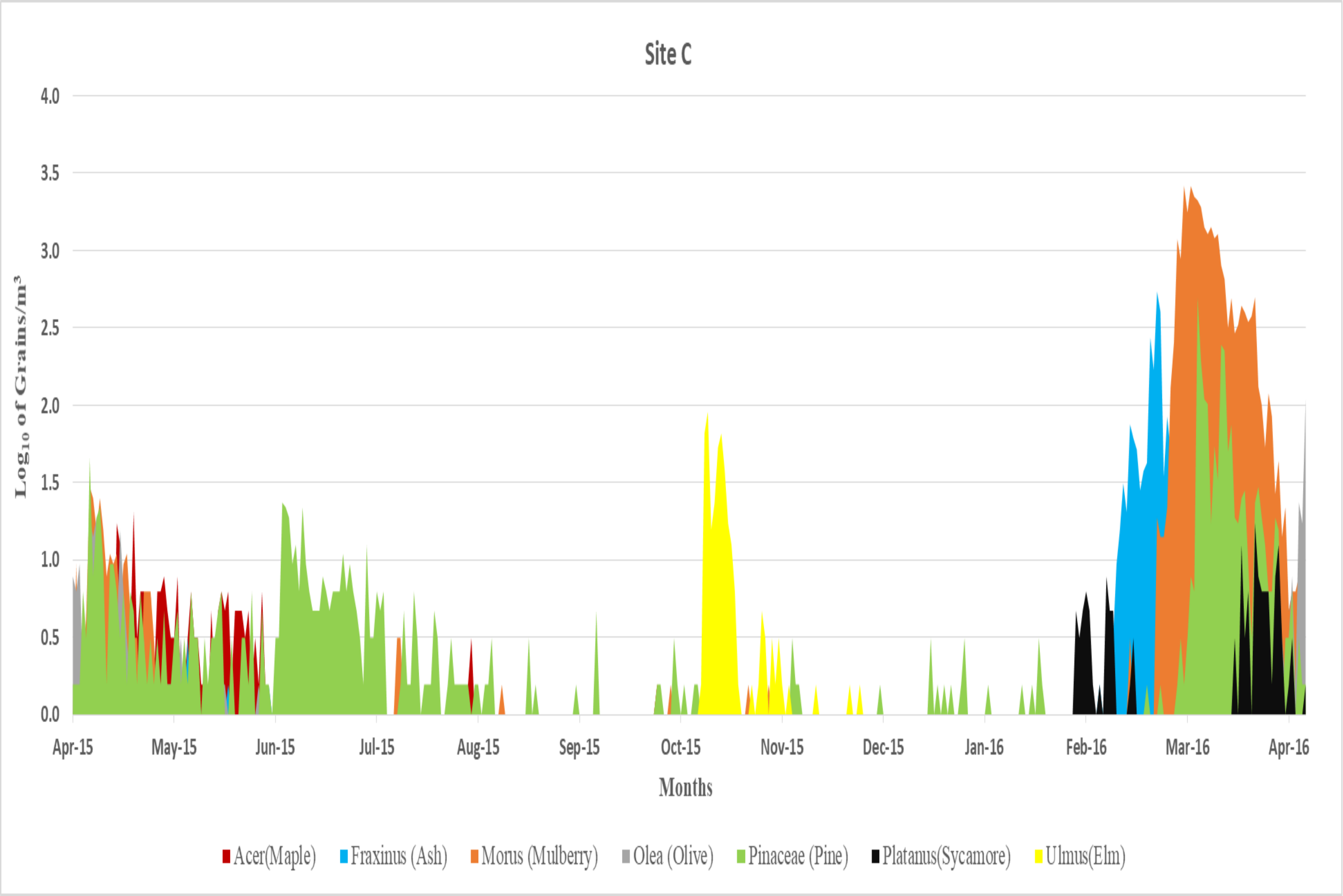

Supplementary Figure 1c. Variation in Tree Pollen for Site C from April 2015 – April 2016.

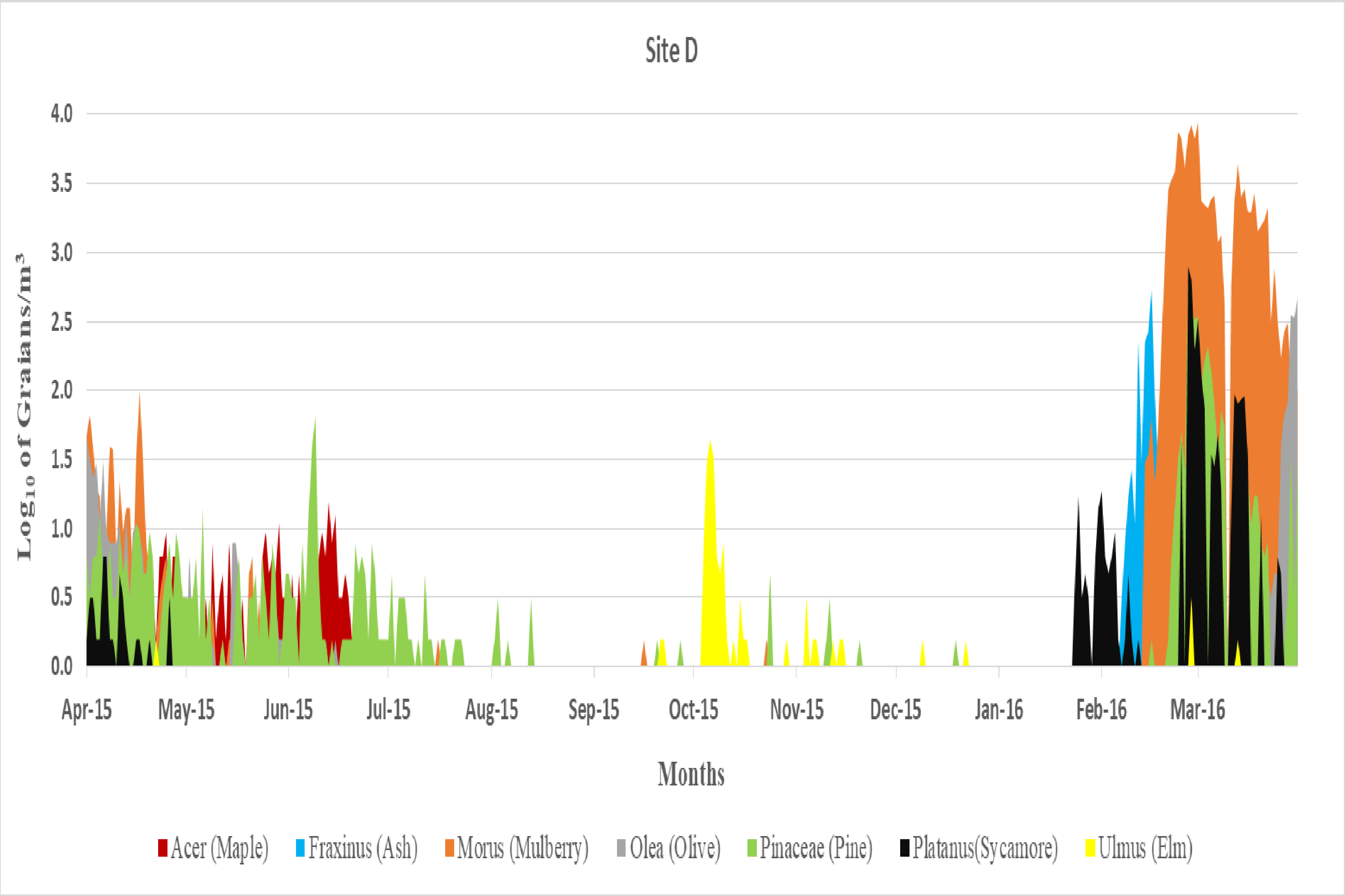

Supplementary Figure 1d. Variation in Tree Pollen for Site D from April 2015 – April 2016.

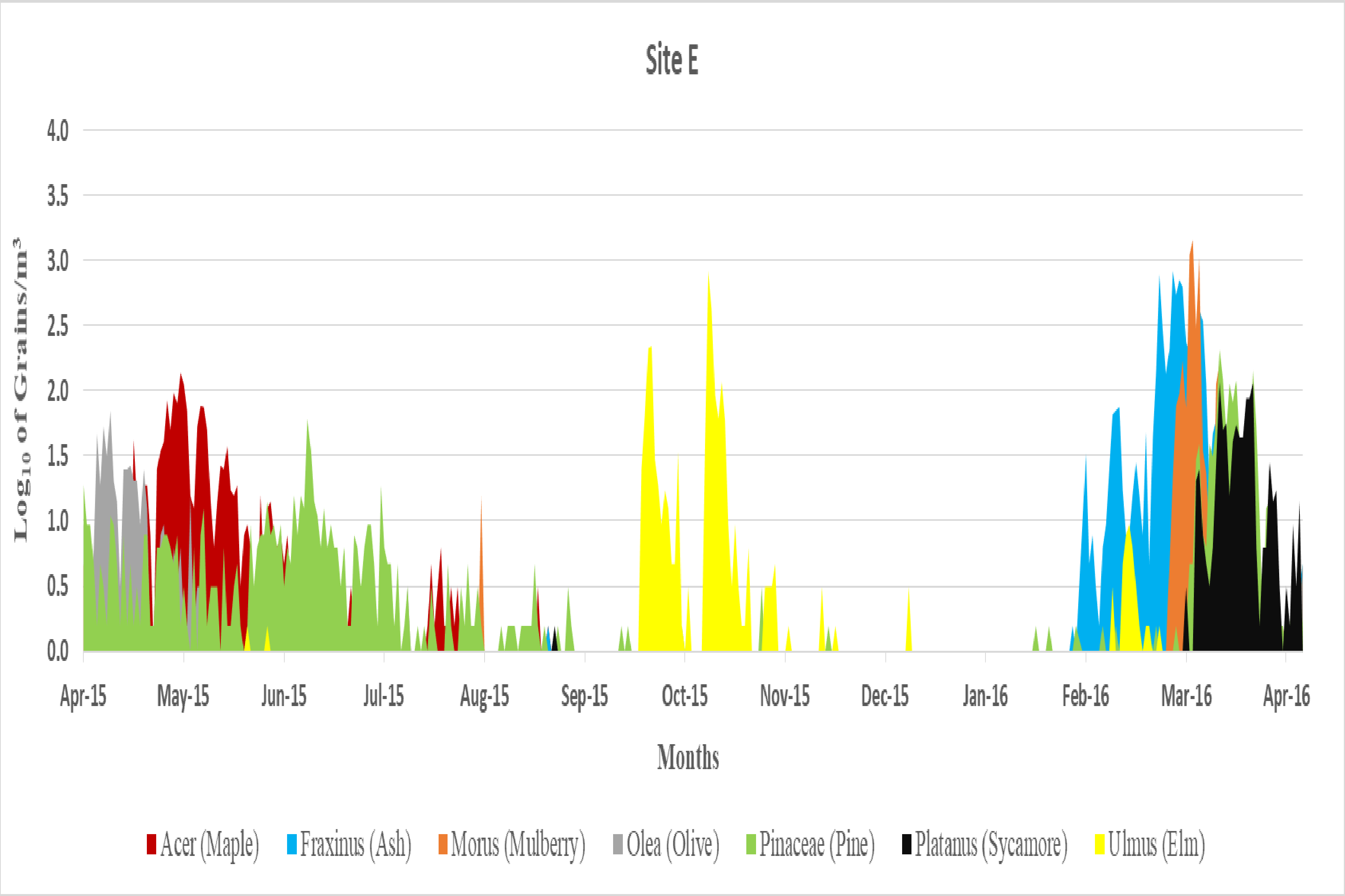

Supplementary Figure 1e. Variation in Tree Pollen for Site E from April 2015 – April 2016.
